# Effects of *Caragana korshinskii* aqueous extract on growth performance, antioxidant capacity, and immune function of sheep under cold stimulation

**DOI:** 10.5194/aab-68-339-2025

**Published:** 2025-05-27

**Authors:** Baoer Pan, Jinyun Yang, Jiuyue Li, Mengzhu Xu, Min Yang, Mengling Peng, Jianbo Cheng, Shuyuan Xue, Juhua Wang

**Affiliations:** 1 College of Veterinary medicine, Anhui Agricultural University, Hefei 230036, China; 2 Inner Mongolia Academy of Agricultural & Animal Husbandry Sciences, Huhhot 00031, China

## Abstract

Cold stimulation can impair the immune and antioxidant functions of animals, which further leads to a decline in animal production performance. This study aimed to investigate the effect of the aqueous extract of *Caragana korshinskii* (CK) on the antioxidant and immunological properties of sheep under oxidative stress induced by cold stimulation. In this study, a total of 54 healthy 2-month-old sheep were randomly divided into six groups, namely the control group and experimental groups I–V. The control group sheep were fed the basal diets, while the sheep in experimental groups I–V had their diets supplemented with 4, 6, 8, 10, and 12 $g\;{\mathrm{kg}}^{-1}$ of CK aqueous extract, respectively; the sheep from all groups were housed in semi-closed enclosures for 65 d (the pre-trial period was 15 d, and the experimental period was 50 d) in a cold environment ( -20 to -10 °C ). The results showed that the final body weight and average daily gain (ADG) of sheep in groups I, II, and III were significantly increased compared with the control group ( p<0.05 ), but there was no significant difference in average daily feed intake (ADFI) ( p>0.05 ). Total antioxidant capacity (T-AOC), glutathione peroxidase (GSH-Px), and superoxide dismutase (SOD) were decreased ( p<0.05 ); malondialdehyde (MDA) was increased ( p<0.05 ); and the tumour necrosis factor- α (TNF- α ), interleukin-2 (IL-2), and interleukin-6 (IL-6) were increased ( p<0.05 ), whereas interleukin-10 (IL-10), immunoglobulin G (IgG), and immunoglobulin M (IgM) were decreased ( p<0.05 ) in the blood of the control sheep after 50 d of cold stimulation. In contrast, T-AOC, GSH-Px, and SOD increased ( p<0.05 ); MDA decreased ( p<0.05 ); and TNF- α , IL-2, and IL-6 decreased ( p<0.05 ), whereas IL-10, IgM, and IgG increased ( p<0.05 ) in the group with the addition of CK aqueous extract. Moreover, the CK aqueous extract decreased MDA ( p<0.05 ) and increased T-AOC, GSH-Px, and SOD in sheep liver ( p<0.05 ) and decreased TNF- α , IL-2, and IL-6 ( p<0.05 ) and increased IL-10, IgM, and IgG in the thymus and spleen ( p<0.05 ). These results suggest that the addition of CK aqueous extract can promote sheep growth and improve oxidative stress, inflammation, and immune response to chronic cold stimulation in sheep.

## 1 Introduction

Cold stimulation is a common phenomenon in livestock and poultry breeding in cold regions in winter, which affects livestock performance and product quality and, more seriously, leads to morbidity and death (Collins and Blackie, 2021; Liu et al., 2021; Tsiouris et al., 2015). At the same time, cold stimulation can cause disorders in terms of self-regulation, oxidative stress, and gradual impairment of the animal's immune system (Venditti et al., 2010; Zhao et al., 2014), which can lead to a decrease in the performance of animal production. It has been demonstrated that cold stimulation activates apoptosis and oxidative stress and releases inflammatory cytokines in mice (Liu et al., 2022), which leads to liver damage. Prolonged cold exposure increases the level of $H_{2}O_{2}$ in the liver while decreasing the levels of superoxide dismutase (SOD) and glutathione peroxidase (GSH-Px), causing oxidative stress in the mouse liver and, further, causing damage to the organism (Xue et al., 2018). Therefore, one of the most effective means of relieving cold stimulation is to improve the animal's immune response and antioxidant function.

Studies have shown that plant extracts contain biologically active substances such as polysaccharides, flavonoids, polyphenols, and saponins, which have antioxidant and immune-boosting properties, promote the production of antioxidant enzymes, and enhance the scavenging of reactive oxygen radicals (Luan et al., 2017; Michalak, 2023). The development and utilization of novel plant-derived antioxidants constitute an important way to alleviate oxidative stress in livestock and poultry. Studies have confirmed that *Caragana korshinskii* (CK) extract contains phenolic bioactive substances such as flavonoids, alkaloids, and triterpenoids (Luan et al., 2017; Zeng et al., 2017), which have antioxidant, immunomodulatory, and growth-promoting effects (Bucciantini et al., 2021; Rattmann et al., 2009). The addition of plant extracts rich in polyphenols (rosemary, grape, citrus, marigold) can limit lipid peroxidation and improve the antioxidant capacity of sheep (Gladine et al., 2007). The polyphenol-rich hazelnut peel dietary supplement helps counteract the inflammation and oxidative stress associated with the growing season of lambs (Ciliberti et al., 2024). A polyphenol compound, the secoisolariciresinol diglucoside extract, optimized rumen microbiota composition and rumen epithelial cell development, promoting growth in pre-weaning lambs (Liu et al., 2025). Some studies have shown that about 70 % of the selection of extractants for plant extracts is focused on organic reagents such as ethanol (Ngibad, 2019), acetone (Panda et al., 2020), *n*-butanol (Ouattar et al., 2022), and chloroform (Nisar et al., 2022). Although organic solvents have lower boiling points and high extraction efficiency, it is difficult to ensure zero residues of organic solvents as feed additives and nutraceuticals, which is a non-negligible problem for the development of safe drug-feeding and homologous feed additives. Moreover, when solvents are used as extractants, the extracted active substances often contain a large number of tannins. This can damage the digestive system and affect the function of nutrient absorption and the growth and development of animals (Sakandar et al., 2019). However, heat treatment can deactivate them by breaking the chemical bonds of tannins (Rajha et al., 2014). The traditional water extraction method can not only remove the anti-nutritional effects of tannins (Ismayati et al., 2024) but also protect human and animal health and reduce the use of antibiotics, thus further providing high-quality livestock and poultry products for humans. However, it is still unclear whether the CK aqueous extract can affect the antioxidant and immune properties of cold-stressed sheep and what their appropriate additional doses are. Therefore, the research objective of this study is to study the effects of different aqueous extracts of CK on the antioxidant properties of sheep serum and liver, as well as on the immunological properties of the serum, thymus, and spleen, and to determine the effects of CK aqueous extract on the antioxidant and immunological properties of sheep in a cold environment and the appropriate dosage. This will provide the basis for the application of CK extract as a new green-feed additive in meat sheep production.

## 2 Materials and methods

### 2.1 CK aqueous extract preparation

The samples of CK were collected at the Siziwang Banner base of the Inner Mongolia Academy of Agricultural and Animal Husbandry Sciences, and the newly emerged whole branches of plants were collected as test samples. The dates of collection were from 25 to 30 May. The CK aqueous extract was extracted by means of a water bath at 100 °C (solid-to-liquid ratio: 1:25 ; extraction time: 120 min), concentrated by rotary evaporation, and freeze-dried. The extraction rate of the water extract was 5 %. The main components of CK aqueous extract were kaferol, quercetin, and glucosinolate ascorbate derivatives, as observed by means of high-performance liquid chromatography–mass spectrometry (HPLC–MS). The relative contents were 28.93 %, 7.52 %, and 0.13 %, respectively.

### 2.2 Experimental animals

The Dumeng crossbred sheep were provided by the Institute of Animal Husbandry of the Inner Mongolia Academy of Agricultural and Animal Husbandry Sciences.

### 2.3 Experimental design

In this experiment, 54 healthy 2-month-old sheep were randomly divided into six groups (control group and experimental groups I–V), with three replicates of three sheep in each group. The control group was fed a basal diet, and the experimental groups I–V were fed diets supplemented with 4, 6, 8, 10, and 12 $g\;{\mathrm{kg}}^{-1}$ of CK aqueous extract in the basal diet, respectively; all sheep were housed in semi-closed enclosures for 65 d (the pre-trial period was 15 d, and the experimental period was 50 d) in a cold environment ( -20 to -10 °C ). The feed formulations used in this experiment were formulated according to the US NRC standard (1998) and commissioned to Hengfu Feed Machinery Co. The basal-diet formulation and nutrient levels of the sheep are shown in Table 1.

**Table 1:** Composition and nutrient levels of basal diets (dry-matter basis %).

Items	Control group	Group I	Group II	Group III	Group IV	Group V
Ingredients						
Cornstalk	25	24.6	24.4	24.2	24	23.8
Corn	45	45	45	45	45	45
Cottonseed meal	8	8	8	8	8	8
Bean meal	7	7	7	7	7	7
SPC	1	1	1	1	1	1
Rapeseed meal	4	4	4	4	4	4
Cane molasses	3	3	3	3	3	3
NaCl	0.6	0.6	0.6	0.6	0.6	0.6
Limestone	1.4	1.4	1.4	1.4	1.4	1.4
${\mathrm{NaHCO}}_{3}$	0.7	0.7	0.7	0.7	0.7	0.7
${\mathrm{CaHPO}}_{3}$	1.2	1.2	1.2	1.2	1.2	1.2
${\mathrm{CaSO}}_{4}$	0.6	0.6	0.6	0.6	0.6	0.6
${\mathrm{NH}}_{4}\mathrm{Cl}$	0.5	0.5	0.5	0.5	0.5	0.5
Premix	2	2	2	2	2	2
*Caragana korshinskii*	0	0.4	0.6	0.8	1	1.2
aqueous extracts						
Total	100	100	100	100	100	100
Nutrient level						
CP	13.77	13.52	13.77	13.55	13.53	13.78
EE	2.66	2.77	2.68	2.93	2.91	2.69
NDF	39.98	39.45	39.66	39.25	39.29	39.38
ADF	18.17	17.70	17.73	17.04	17.48	17.63
Lignin	2.66	2.86	2.44	2.71	2.49	2.18
Ash	7.72	7.63	7.31	7.18	7.79	7.33
Ca	1.08	0.98	1.06	1.35	1.33	1.38
P	0.57	0.46	0.54	0.56	0.57	0.58

Note that SPC denotes soy protein concentrate, NaCl denotes salt, ${\mathrm{NaHCO}}_{3}$ denotes baking soda, CaHPO ${}_{3}$ denotes calcium hydrogen phosphate, ${\mathrm{CaSO}}_{4}$ denotes calcium sulfate, ${\mathrm{NH}}_{4}\mathrm{Cl}$ denotes ammonium chloride, CP denotes crude protein, EE denotes crude fat, NDF denotes neutral detergent fibre, ADF denotes acid detergent fibre, ash denotes ash content, Ca denotes calcium, and P denotes phosphorus.

### 2.4 Determination of growth performance

Each sheep was weighed at the beginning and end of the trial period, and the feed intake of each group was recorded. The average daily gain (ADG) was calculated by dividing the weight of each group by the number of trial days and sheep, the average daily feed intake (ADFI) was calculated by dividing the total feed intake of each group by the number of trial days and sheep, and the feed conversion ratio (FCR) was calculated by dividing the average daily feed intake by the average daily gain.

### 2.5 Sample collection

On the 1st and 50th days of the positive feeding period, 10 mL of blood was collected from the jugular vein using an anticoagulant-free blood collection tube before the morning feeding, and, after the blood was left to stand for 20 min, it was centrifuged at 3000 $r\;\min^{-1}$ for 20 min, and then the serum was collected and preserved at -20 °C for antioxidant and immune index tests. At the end of the experiment, three sheep in each group were randomly selected for slaughter, and the liver, thymus, and spleen were collected, immediately homogenized, and stored in a refrigerator at -80 °C . The collected liver was used for the determination of oxidative stress indexes. The thymus and spleen were used for the determination of immune indexes. To minimize the test error, each tissue sample was separately collected from the same site.

### 2.6 Determination of oxidative stress indexes

The tissue was removed from the -80 °C refrigeration, weighed according to the weight (g) / PBS volume (mL) = 1/9 formula, mechanically homogenized on ice to prepare 10 % tissue homogenate, and centrifuged at 4 °C and 3000 $r\;\min^{-1}$ for 10 min, and then the supernatant was taken to be measured. The serum of each group of sheep at days 1 and 50 and the liver homogenates at the end of the experiment were collected, and the contents of malondialdehyde (MDA), total antioxidant capacity (T-AOC), SOD, and GSH-Px were determined according to the corresponding assay kits (Beijing Sinoco Biotechnology Research Institute, Beijing, China), and the experimental methods were carried out by referring to the specific operation steps of the kits. The experimental data were tested three times.

### 2.7 Determination of immunity indexes

The serum of each group of sheep on the 1st and 50th days of preservation and the thymus and spleen homogenates at the end of the experiment were collected, and the serum, thymus, and spleen interleukin-2 (IL-2), interleukin-6 (IL-6), interleukin-10 (IL-10), tumour necrosis factor- α (TNF- α ), immunoglobulin G (IgG), and immunoglobulin M (IgM) were determined according to the corresponding test kits (Nanjing Jiancheng Bioengineering Institute, Nanjing, China). The test methods and procedures were performed according to the instructions of the corresponding enzyme immunoassay kits. The experimental data were tested three times.

### 2.8 Data analysis

The difference significance of the data was analysed by means of the one-way ANOVA and paired t -test methods of the IBM SPSS Statistics 25.0 software and was expressed as means ± standard error of means (means ± SEM), with p<0.01 being highly significant, p<0.05 being significant, and p>0.05 being non-significant.

## 3 Results

### 3.1 Effects of CK aqueous extract on growth performance of sheep

The effects of CK aqueous extract on the growth performance of sheep are shown in Table 2. Dietary CK aqueous extract had significant effects on the final body weight, ADG, and FCR of the sheep ( p<0.05 ) but had no significant effects on the ADFI of the sheep ( p>0.05 ). The final body weight of groups I, II, and III was significantly higher than that of the other groups ( p<0.05 ) and was increased by 13.07 %, 15.27 %, and 15.94 %, respectively, compared to the control group. The final body weight of the control group was significantly higher than that of group V ( p<0.05 ). The ADG of sheep in groups I, II, and III was significantly higher than that in the other groups ( p<0.05 ) and was increased by 21.77 %, 33.92 %, and 32.34 %, respectively, compared with the control group. The ADG in the control group was significantly higher than that in groups IV and V ( p<0.05 ). The FCR in groups IV and V was significantly higher than that in the other groups ( p<0.05 ).

**Table 2:** Effects of CK aqueous extract on growth performance of sheep ( n=9 ).

Items	Control group	Group I	Group II	Group III	Group IV	Group V
IBW (kg)	20.92±0.74	21.47±1.10	22.20±0.68	22.83±1.35	21.99±0.56	21.13±0.80
FBW (kg)	29.61±2.44 b	33.48±1.96 a	34.13±2.79 a	34.33±1.96 a	28.44±1.05 bc	26.79±1.40 c
ADG ( $g\;d^{-1}$ )	173.80±56.37 b	211.63±37.20 a	232.75±47.93 a	230.00±27.06 a	133.43±26.55 c	111.71±12.98 c
ADFI ( $g\;d^{-1}$ )	1310.40±187.25	1600.40±246.85	1590.41±216.73	1655.34±251.46	1395.42±179.73	1315.85±188.19
FCR	6.93±0.35 b	7.56±0.01 b	6.83±0.01 b	7.19±0.02 b	10.75±0.14 a	11.77±0.03 a

In the same row, values with different small letters denote a significant difference ( p<0.05 ), while the small letters or no letters mean no significant difference ( p>0.05 ). Abbreviations: IBW denotes initial body weight, FBW denotes final body weight, ADG denotes average daily gain, ADFI denotes average daily feed intake, and FCR denotes feed conversion rate.

### 3.2 Effects of CK aqueous extract with different contents on antioxidant properties of sheep

#### 3.2.1 Effects of CK aqueous extract on serum oxidative stress indexes of sheep during different feeding periods

**Figure 1:**
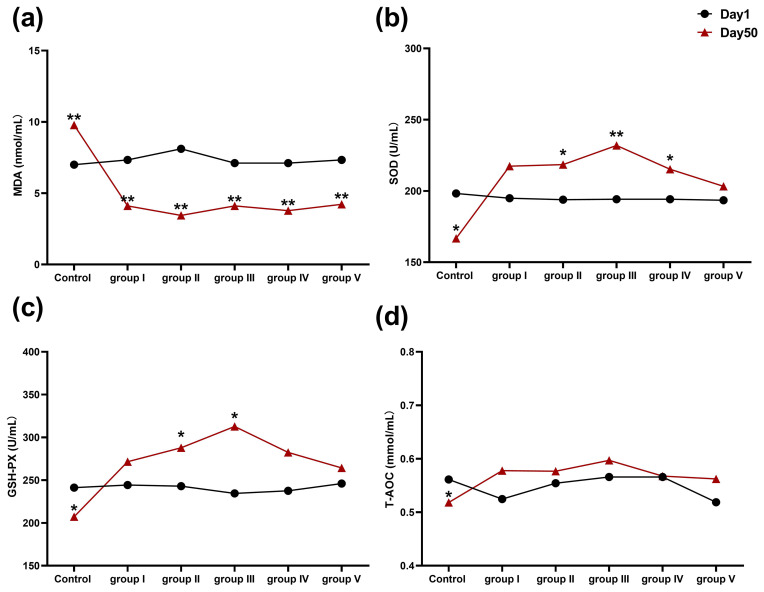
Effect of aqueous extract of CK on the levels of MDA, SOD, GSH-Px, and T-AOC in the serum of sheep during different feeding periods. Note that the control group was fed a basal diet, and the experimental groups I–V were fed basal diets supplemented with 4, 6, 8, 10, and 12 $g\;{\mathrm{kg}}^{-1}$ of CK aqueous extract. No mark indicates that the difference is not significant ( p>0.05 ), * indicates that the difference is significant ( p<0.05 ), and ** indicates that the difference is highly significant ( p<0.01 ). MDA denotes malondialdehyde, SOD denotes superoxide dismutase, GSH-Px denotes glutathione peroxidase, and T-AOC denotes total antioxidant capacity.

The effects of CK aqueous extract on the serum oxidative stress indexes of sheep during different feeding periods are shown in Fig. 1. The serum MDA content of the sheep in the control group was highly significantly higher at 50 d than at 1 d ( p<0.01 ), and the serum MDA content of the remaining treatment groups (experimental groups I–V) was highly significantly lower at 50 d than at 1 d ( p<0.01 ) (Fig. 1a). SOD activity in the control group at 50 d was significantly lower than that at 1 d ( p<0.05 ), whereas, at 50 d, SOD activity in sheep serum in experimental groups II ( p<0.05 ), III ( p<0.01 ), and IV ( p<0.05 ) was significantly higher than that at 1 d (Fig. 1b). GSH-Px activity in sheep serum was significantly lower in the control group at 50 d than at 1 d ( p<0.05 ), whereas, at 50 d, GSH-Px activity in sheep serum was significantly higher in experimental groups II and III than at 1 d ( p<0.05 ) (Fig. 1c). The serum T-AOC level of sheep in the control group on day 50 was significantly lower than that on day 1 ( p<0.05 ) (Fig. 1d). In summary, we found that CK aqueous extract was able to increase the antioxidant capacity of sheep in cold environments.

#### 3.2.2 Effects of CK aqueous extract with different contents on serum oxidation indexes of sheep

**Figure 2:**
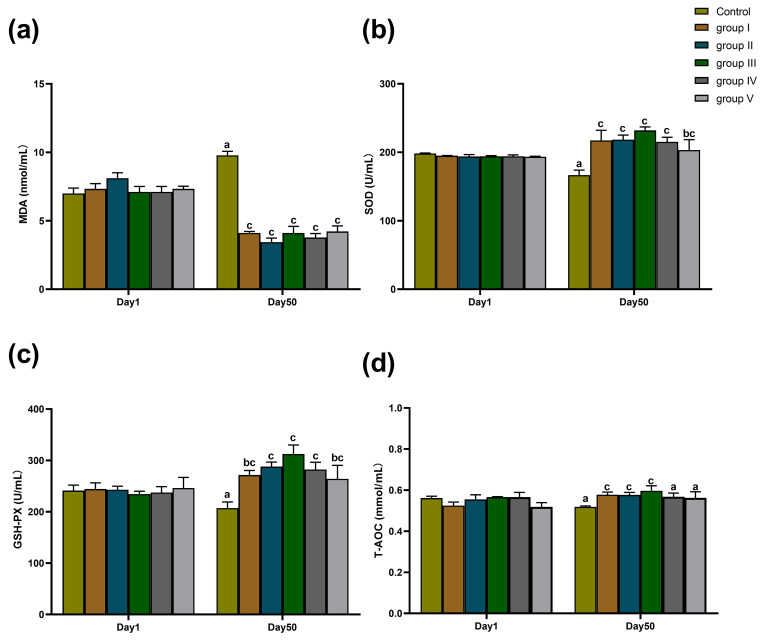
Effects of aqueous extract of CK with different contents on the levels of MDA, SOD, GSH-Px, and T-AOC in the serum of the sheep in each group. Note that the control group was fed a basal diet, and the experimental groups I–V were fed basal diets supplemented with 4, 6, 8, 10, and 12 $g\;{\mathrm{kg}}^{-1}$ of CK aqueous extract. Data labelled without markers or shown with the same letter indicate insignificant differences ( p>0.05 ), adjacent letters indicate significant differences ( p<0.05 ), and intermediate letters indicate highly significant differences ( p<0.01 ). MDA denotes malondialdehyde, SOD denotes superoxide dismutase, GSH-Px denotes glutathione peroxidase, and T-AOC denotes total antioxidant capacity.

The effects of CK aqueous extract with different contents on the serum oxidation indexes of sheep are shown in Fig. 2. As shown in Fig. 2a–d, there were no significant differences in terms of MDA content, T-AOC level, SOD, and GSH-Px activities in the serum of sheep among all groups on day 1 of the experiment period ( p>0.05 ). After 50 d of the experiment, the MDA contents in the serum of sheep in experimental groups I–V were all significantly lower than that of the control group ( p<0.05 ) (Fig. 2a). SOD activity in the serum of sheep in experimental groups I, II, III, IV ( p<0.01 ), and V ( p<0.05 ) was significantly higher than that of the control group (Fig. 2b). GSH-Px activity in the serum of sheep in experimental groups I ( p<0.05 ), II ( p<0.01 ), III ( p<0.01 ), IV ( p<0.01 ), and V ( p<0.05 ) was significantly higher than that of the control group (Fig. 2c). The serum levels of T-AOC in sheep from groups I, II, and III were significantly higher than those in the control group and groups IV and V ( p<0.01 ) (Fig. 2d).

#### 3.2.3 Effects of CK aqueous extract with different contents on liver oxidation indexes of sheep

**Figure 3:**
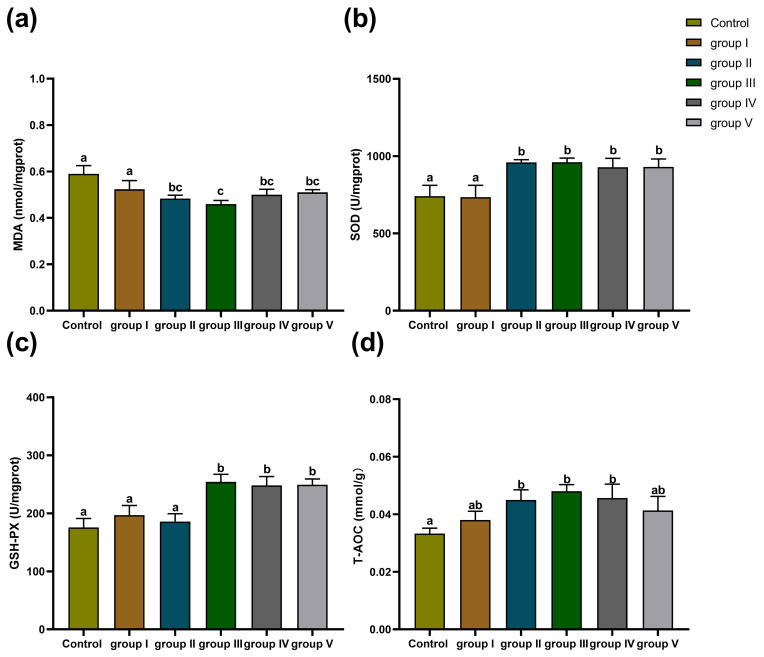
Effect of different contents of aqueous extract of CK on the levels of MDA, SOD, GSH-Px, and T-AOC in sheep liver. Note that the control group was fed a basal diet, and the experimental groups I–V were fed basal diets supplemented with 4, 6, 8, 10, and 12 $g\;{\mathrm{kg}}^{-1}$ of CK aqueous extract. Data labelled without markers or shown with the same letter indicate insignificant differences ( p>0.05 ), adjacent letters indicate significant differences ( p<0.05 ), and intermediate letters indicate highly significant differences ( p<0.01 ). MDA denotes malondialdehyde, SOD denotes superoxide dismutase, GSH-Px denotes glutathione peroxidase, and T-AOC denotes total antioxidant capacity.

The effects of CK aqueous extract with different contents on the liver oxidation indexes of sheep are shown in Fig. 3. The MDA levels of the sheep livers in the control group and group I were significantly higher than those in groups II, III, IV, and V ( p<0.05 ) (Fig. 3a). The SOD activity of the sheep livers in the control group and group I was significantly lower than that in groups II, III, IV, and V ( p<0.05 ) (Fig. 3b). The GSH-Px activity of the sheep livers in the control group and groups I and II was significantly lower than that in groups III, IV, and V ( p<0.05 ) (Fig. 3c). The levels of T-AOC in the sheep livers in the control group were significantly lower than those in groups II, III, and IV ( p<0.05 ) (Fig. 3d). In summary, we found that CK aqueous extract can improve the antioxidant capacity of sheep in a cold environment, especially with the addition of 8 $g\;{\mathrm{kg}}^{-1}$ .

### 3.3 Effects of CK aqueous extract with different contents on the immune performance of sheep

#### 3.3.1 Effect of CK aqueous extract on serum immune indexes of sheep during different feeding periods

**Figure 4:**
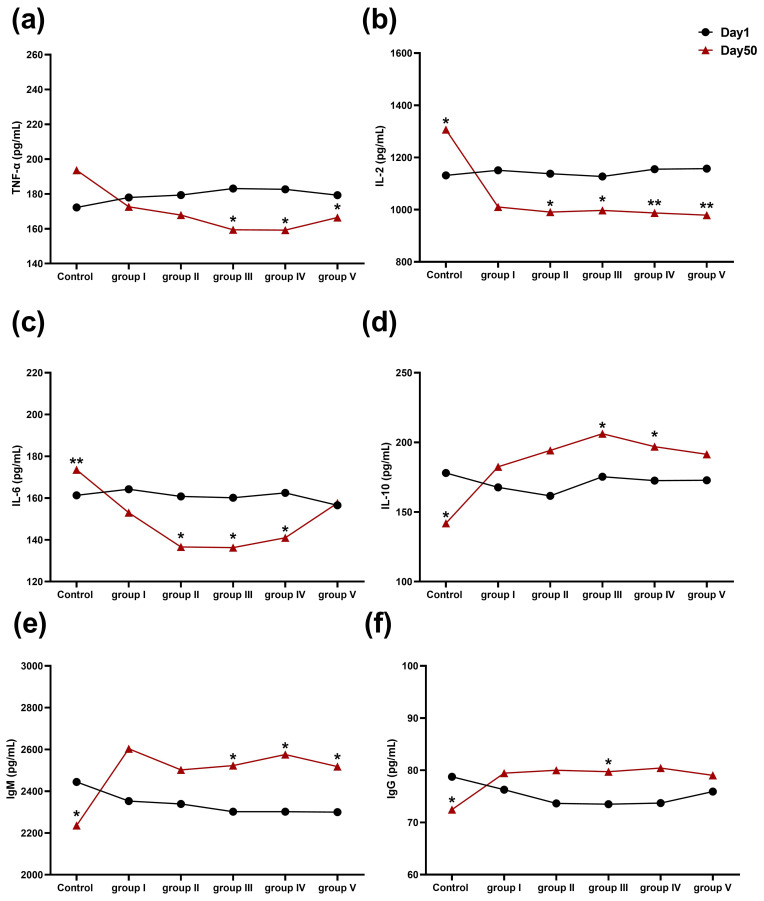
Effects of aqueous extract of CK on the levels of TNF- α , IL-2, IL-6, IL-10, IgM, and IgG in the serum of the sheep in each group during different experimental periods. Note that the control group was fed a basal diet, and the experimental groups I–V were fed basal diets supplemented with 4, 6, 8, 10, and 12 $g\;{\mathrm{kg}}^{-1}$ of CK aqueous extract. No mark indicates that the difference is not significant ( p>0.05 ), * indicates that the difference is significant ( p<0.05 ), and ** indicates that the difference is highly significant ( p<0.01 ). IL-2 denotes interleukin-2, IL-6 denotes interleukin-6, IL-10 denotes interleukin-10, TNF- α denotes tumour necrosis factor- α , IgG denotes immunoglobulin G, and IgM denotes immunoglobulin M.

The effect of CK aqueous extract on the serum immune indexes of sheep during different feeding periods is shown in Fig. 4. After 50 d of the experiment, TNF- α concentrations in the serum of sheep in groups III, IV, and V were significantly lower than those at 1 d ( p<0.05 ) (Fig. 4a). The serum levels of IL-2 in control group sheep were significantly higher than those at 1 d ( p<0.05 ), whereas the serum levels of IL-2 in experimental groups II, III ( p<0.05 ), IV, and V ( p<0.01 ) were significantly lower than those at 1 d. (Fig. 4b). The serum levels of IL-6 in the control group of sheep were very significantly higher than those at 1 d ( p<0.01 ), and the serum levels of IL-6 in the sheep of groups II, III, and IV were significantly lower than those at 1 d ( p<0.05 ) (Fig. 4c). IL-10 concentrations in the serum of control group sheep were significantly lower than those at 1 d ( p<0.05 ), while those in groups III and IV were significantly higher than those at 1 d ( p<0.05 ) (Fig. 4d). The concentrations of IgM in the serum of groups III and V were significantly higher at 50 d than at 1 d ( p<0.05 ) (Fig. 4e). The IgG concentration in the serum of the control group was significantly lower than that at 1 d ( p<0.05 ), whereas the IgG concentration of the sheep serum in group III of the experiment was significantly higher than that at 1 d ( p<0.05 ) (Fig. 4f). In summary, we found that CK aqueous extract was able to improve the immune performance of sheep in cold environments.

#### 3.3.2 Effects of CK aqueous extract with different contents on serum immune indexes of sheep

**Figure 5:**
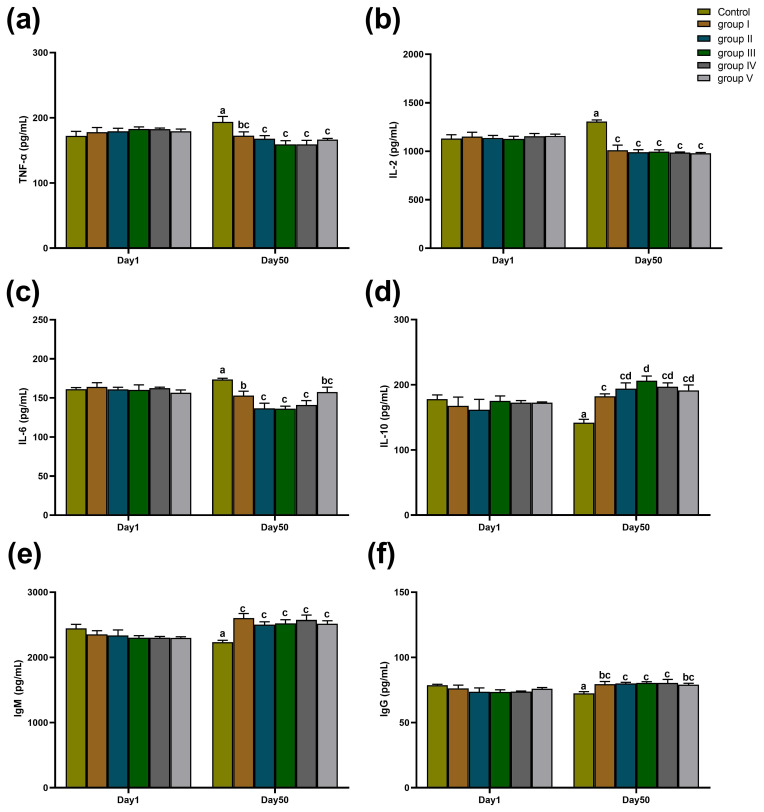
Effect of different contents of aqueous extract of CK on the levels of TNF- α , IL-2, IL-6, IL-10, IgM, and IgG in the serum of sheep in each group. Note that the control group was fed a basal diet, and the experimental groups I–V were fed basal diets supplemented with 4, 6, 8, 10, and 12 $g\;{\mathrm{kg}}^{-1}$ of CK aqueous extract. Data labelled without markers or shown with the same letter indicate insignificant differences ( p>0.05 ), adjacent letters indicate significant differences ( p<0.05 ), and intermediate letters indicate highly significant differences ( p<0.01 ). IL-2 denotes interleukin-2, IL-6 denotes interleukin-6, IL-10 denotes interleukin-10, TNF- α denotes tumour necrosis factor- α , IgG denotes immunoglobulin G, and IgM denotes immunoglobulin M.

The effects of CK aqueous extract with different contents on the serum immune indexes of sheep are shown in Fig. 5. As shown in Fig. 5a–f, there were no significant differences in the serum levels of TNF- α , IL-2, IL-6, IL-10, IgM, and IgG among all groups at day 1 of the experiment period ( p>0.05 ). At day 50 of the experiment period, TNF- α concentrations in the serum of control sheep were significantly higher than those in groups I ( p<0.05 ), II, III, IV, and V ( p<0.01 ) (Fig. 5a). IL-2 levels in the serum of sheep in the control group were all extremely significantly higher than those in groups I, II, III, IV, and V ( p<0.01 ) (Fig. 5b). IL-6 levels in the serum of control sheep were significantly higher than those of groups I ( p<0.05 ), II ( p<0.01 ), III ( p<0.01 ), IV ( p<0.01 ), and V ( p<0.05 ); furthermore, IL-6 levels in the serum of group I were also significantly higher than those of groups II, III, and IV ( p<0.05 ) (Fig. 5c). IL-10 concentrations in the serum of control group sheep were highly significantly lower than those in groups I, II, III, IV, and V ( p<0.01 ), and IL-10 concentrations in the serum of sheep in group III were significantly higher than those in group I ( p<0.05 ) (Fig. 5d). IgM concentrations in the serum of control group sheep were all extremely significantly lower than those of experimental groups I, II, III, IV, and V ( p<0.01 ) (Fig. 5e). The concentration of IgG in the serum of control group sheep was significantly lower than that of groups I ( p<0.05 ), II ( p<0.01 ), III ( p<0.01 ), IV ( p<0.01 ), and V ( p<0.05 ) (Fig. 5f).

#### 3.3.3 Effects of CK aqueous extract with different contents on spleen immune indexes of sheep

**Figure 6:**
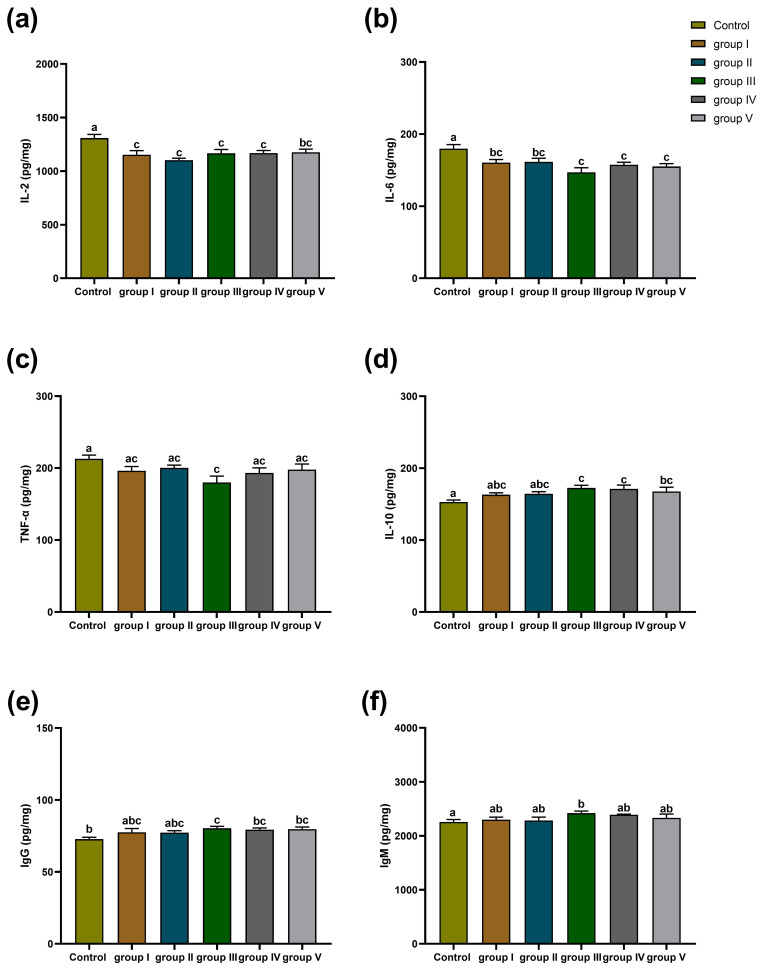
Effect of different contents of aqueous extract of CK on the levels of IL-2, IL-6, IL-10, TNF- α , IgG, and IgM in sheep spleens. Note that the control group was fed a basal diet, and the experimental groups I–V were fed basal diets supplemented with 4, 6, 8, 10, and 12 $g\;{\mathrm{kg}}^{-1}$ of CK aqueous extract. Data labelled without markers or shown with the same letter indicate insignificant differences ( p>0.05 ), adjacent letters indicate significant differences ( p<0.05 ), and intermediate letters indicate highly significant differences ( p<0.01 ). IL-2 denotes interleukin-2, IL-6 denotes interleukin-6, IL-10 denotes interleukin-10, TNF- α denotes tumour necrosis factor- α , IgG denotes immunoglobulin G, and IgM denotes immunoglobulin M.

The effects of CK aqueous extract with different contents on the spleen immune indexes of sheep are shown in Fig. 6. The IL-2 of the spleens in the control group sheep was significantly higher than that in groups I, II, III, IV ( p<0.01 ), and V ( p<0.05 ) (Fig. 6a). The IL-6 content in the spleens of control group sheep was significantly higher than that of groups I, II ( p<0.05 ), III, IV, and V ( p<0.01 ) (Fig. 6b). The TNF- α content in the spleens of control group sheep was highly significantly higher than that of group III ( p<0.01 ) (Fig. 6c). The IL-10 content of the spleens in the control group sheep was significantly lower than that in groups III, IV, and V ( p<0.01 ) (Fig. 6d). The IgG content of the spleens in the control group sheep was significantly lower than that in groups III ( p<0.01 ), IV ( p<0.05 ), and V ( p<0.05 ) (Fig. 6e). The concentration of IgM for the spleens in the control group sheep was significantly lower than that in group III ( p<0.05 ) (Fig. 6f).

#### 3.3.4 Effects of CK aqueous extract with different contents on thymus immune indexes of sheep

**Figure 7:**
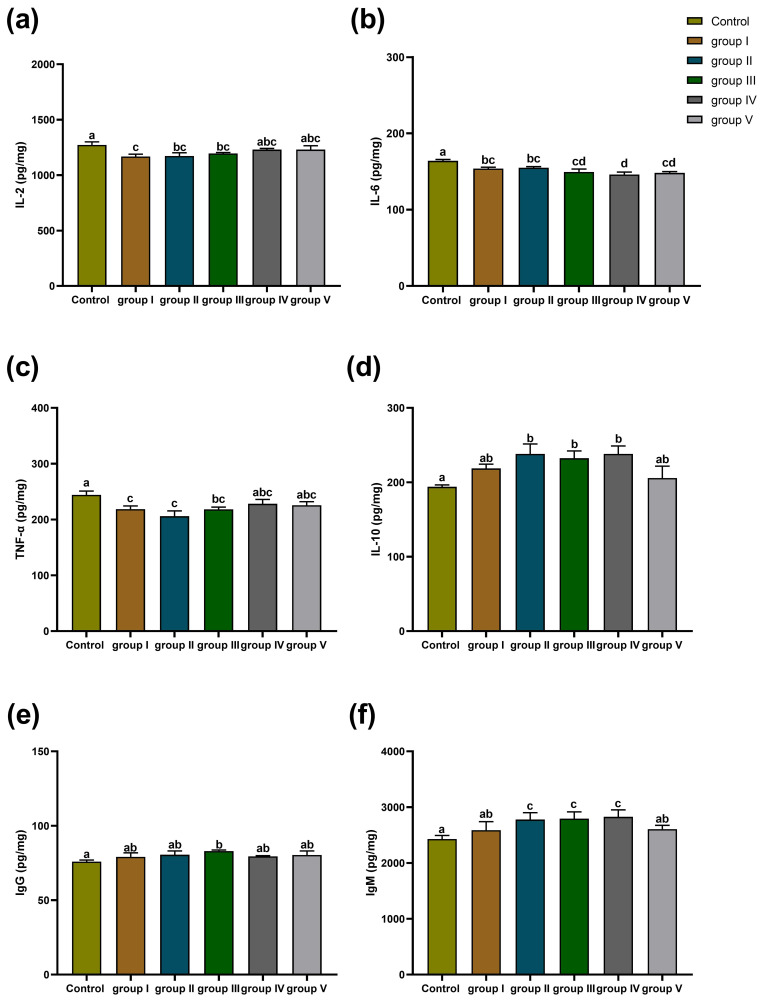
Effect of different contents of aqueous extract of CK on the levels of IL-2, IL-6, IL-10, TNF- α , IgG, and IgM in the thymuses of sheep. Note that the control group was fed a basal diet, and the experimental groups I–V were fed basal diets supplemented with 4, 6, 8, 10, and 12 $g\;{\mathrm{kg}}^{-1}$ of CK aqueous extract. Data labelled without markers or shown with the same letter indicate insignificant differences ( p>0.05 ), adjacent letters indicate significant differences ( p<0.05 ), and intermediate letters indicate highly significant differences ( p<0.01 ). IL-2 denotes interleukin-2, IL-6 denotes interleukin-6, IL-10 denotes interleukin-10, TNF- α denotes tumour necrosis factor- α , IgG denotes immunoglobulin G, and IgM denotes immunoglobulin M.

The effects of CK aqueous extract with different contents on the thymus immune indexes of sheep are shown in Fig. 7. The IL-2 concentration of the thymuses in the control group sheep was significantly higher than that in groups I ( p<0.01 ), II ( p<0.05 ), and III ( p<0.05 ) (Fig. 7a). The IL-6 content of the thymuses in the control group sheep was highly significantly higher than that in groups I, II ( p<0.05 ), III, IV, and V ( p<0.01 ) and was significantly higher in group IV than in groups I and II ( p<0.05 ) (Fig. 7b). The concentration of TNF- α in the thymuses of the control group sheep was significantly higher than that in groups I ( p<0.01 ), II ( p<0.01 ), and III ( p<0.05 ) (Fig. 7c). The concentration of IL-10 in the thymuses of the control group sheep was significantly lower than that in experimental groups II, III, and IV ( p<0.05 ) (Fig. 7d). The IgG content of the thymuses of the control group sheep was significantly lower than that in group III ( p<0.05 ) (Fig. 7e). The IgM content of the thymuses in the control group sheep was highly significantly lower than that in groups II, III, and IV ( p<0.01 ), whereas that in groups I and V was significantly lower than that in groups II, III, and IV ( p<0.05 ) (Fig. 7f).

## 4 Discussion

Cold stress is a physical environmental stressor that affects neuroendocrine, reproductive, and cardiovascular systems and alters biochemical processes in organisms, leading to oxidative damage, apoptosis, and other physiological or pathological responses that can affect the performance, health, and welfare of livestock and poultry, which has a significant impact on the livestock and poultry industry (Collins and Blackie, 2021; Liu et al., 2021; Tsiouris et al., 2015). Dietary plant extracts can promote the growth of livestock and poultry. Studies have shown that the addition of 30 $\mathrm{mg}\;{\mathrm{kg}}^{-1}$ ellagic acid regulated rumen microbiota, enhanced antioxidant capacity, and improved daily weight gain (Zhang et al., 2024). The addition of *Astragalus membranaceus* root supplement in the basal diet could improve the average daily gain and feed conversion rate of Tibetan sheep (Wang et al., 2021). Dietary supplementation with 1 $g\;{\mathrm{kg}}^{-1}$ compound plant additive (mainly composed of Astragalus and Codonginseng) does not affect the ADFI of growing-finishing pigs but can significantly increase the final weight and ADG of growing-finishing pigs during the entirety of the experiment period (Cheng et al., 2020). Adding 20 $g\;{\mathrm{kg}}^{-1}$ of grape seeds to the base diet can increase final body weight and average daily gain of broilers, can improve feed conversion, and does not affect feed intake (Abu Hafsa and Ibrahim, 2018). The results of this study showed that, compared with the control group, dietary supplementation with 4, 6, and 8 $g\;{\mathrm{kg}}^{-1}$ of CK aqueous extract can significantly increase the final body weight and ADG of sheep but has no significant effect on ADFI. With the addition level of CK aqueous extract being increased to 10–12 $g\;{\mathrm{kg}}^{-1}$ , the growth performance of sheep decreased, indicating that the growth performance of sheep was affected by the addition level of CK aqueous extract.

Studies have shown that plant extracts contain biologically active substances such as polysaccharides, flavonoids, polyphenols, and saponins, which have antioxidant and immune-boosting properties, promote the production of antioxidant enzymes, and enhance the scavenging of reactive oxygen radicals (Luan et al., 2017; Michalak, 2023). In recent years, studies have found that CK mallow contains important active components such as polyphenols (Luan et al., 2016; Zeng et al., 2017; Zhou et al., 2016). We analysed the active components of the CK aqueous extract by means of high-performance liquid chromatography–mass spectrometry (HPLC–MS) and found that the aqueous extract mainly consisted of 12 polyphenolic actives, such as kaempferol, quercetin, and derivatives of ascorbic acid. Phenolic compounds have a variety of bioactive effects, including antioxidant, antiallergic, anti-inflammatory, and antimicrobial activities (Laganà et al., 2019; Ulanowska and Olas, 2021). Lambs fed grape residue rich in plant polyphenols had significantly increased catalase (CAT) and GSH content in their blood and tissues and showed significantly increased antioxidant mechanisms (Kafantaris et al., 2017). The natural plant antitoxin polyphenol resveratrol can enhance the antioxidant enzymes (GSH-Px and CAT) of ewes at different reproductive stages to relieve oxidative stress and improve the health of the ewes (Li et al., 2024). Moreover, in the treatment of septic mice, kaempferol also inhibits oxidative stress by increasing the activity of the antioxidant enzymes SOD and catalase, as well as the non-enzymatic antioxidant GSH (Rabha et al., 2018). Studies have shown that flavonoids from plant extracts can reduce MDA levels and increase the activities of SOD, GSH, and GSH-Px in rats with hepatic fibrosis, thereby reducing lipid peroxidation and attenuating hepatocyte damage in rats (Guo et al., 2017). Therefore, in the present study, the CK aqueous extract was chosen to study its effect on the antioxidant and immune properties of the organisms of sheep under cold stimulation.

MDA is the end product of the peroxidation reaction of free radicals acting on lipids and is often used as a marker of the occurrence of oxidative stress in the body (Fei et al., 2016). Therefore, in the present study, MDA was used as an indicator of oxidative stress in sheep organisms under cold environmental stimuli, and it was found that, with the prolongation of cold-stress time, the MDA content in the serum of sheep without the addition of CK aqueous extract increased significantly, indicating that oxidative stress occurs in sheep organisms in cold environments. However, the addition of a CK aqueous extract was able to reduce the MDA content in sheep serum, suggesting that the addition of a CK aqueous extract could alleviate cold stress and oxidative stress in the sheep organism. SOD, GSH-Px, and T-AOC are important antioxidant indices in the body (Yang et al., 2023a). Among them, SOD catalyses the decomposition of harmful superoxide anions into molecular oxygen, which is then converted into hydrogen peroxide (Xiao et al., 2024). GSH-PX can reduce toxic peroxides to non-toxic hydroxyl compounds while promoting the decomposition of $H_{2}O_{2}$ , thus protecting the structure and function of cell membranes from interference and damage by peroxides (Porfire et al., 2014). T-AOC levels represent the cumulative effect of all antioxidants, including both enzymatic and non-enzymatic compounds, and, thus, these metrics can be used as a comprehensive parameter reflecting the total antioxidant capacity of an organism (Xiao et al., 2024). Therefore, we used GSH-Px, SOD, and T-AOC as measures of the changes in the oxidative properties of the organisms in this study. It was found that T-AOC level, SOD, and GSH-Px activity in the serum of sheep in the group without added CK aqueous extract were significantly reduced with prolonged rearing time in the cold environment. The SOD and GSH-Px activities were significantly increased after the addition of CK aqueous extract, indicating that the addition of CK aqueous extract to the feed could result in resistance to the occurrence of lipid peroxidation caused by cold stimulation; in particular, the best effect was achieved with the addition of 8 $g\;{\mathrm{kg}}^{-1}$ .

The liver plays an important role in chronic cold exposure as the largest glycogen storage organ in the body and as the main heat-producing organ during quiet times (Liu et al., 2022). When the body is exposed to a cold environment, the metabolic rate of the liver increases, and the production of reactive oxygen species (ROSs) increases with enhanced mitochondrial energy metabolism, which disrupts the balance of the oxidative–antioxidant system and leads to oxidative stress (Sahin and Gümüşlü, 2004). Therefore, the present study investigated the effect of the addition of CK aqueous extract on the oxidative properties of the liver. It was found that the highest MDA content and the lowest SOD, GSH-Px, and T-AOC activities were found in the livers of sheep without the addition of CK aqueous extract under cold-stimulation conditions. In contrast, the addition of CK aqueous extract significantly reduced the MDA content in the liver and increased the levels of SOD, GSH-Px activity, and T-AOC; the effect was most pronounced with the addition of 6–12 $g\;{\mathrm{kg}}^{-1}$ to the diets. This suggests that cold stimulation caused lipid peroxidation in sheep liver, which could be broken down by the addition of CK aqueous extract to the feed, depending on the amount of CK aqueous extract added.

In conclusion, adding a suitable amount of CK aqueous extract to the diet can improve the overall antioxidant capacity of sheep under cold stimulation. In particular, the best effect was achieved with the addition of 8 $g\;{\mathrm{kg}}^{-1}$ .

When livestock and poultry suffer from cold stress, their bodies will undergo a variety of complex abnormal reactions, with the immune system and other functions being affected. Studies have shown that cold exposure affects immune system function (Hangalapura et al., 2006; Shi et al., 2022). Cytokines are important information molecules of the immune system (Lin and Karin, 2007) which play an indispensable role in the immune response process of the body. During the inflammatory process, as the relevant immune cells (lymphocytes, monocytes–macrophages, etc.) are over-activated, a large number of cytokines are produced, and changes in their levels can reflect the presence of an inflammatory response (Kunnumakkara et al., 2021). Among them, IL-2 and IL-6 are T-cell growth factors and B-cell growth factors, respectively, which are major pro-inflammatory factors in the inflammatory response and play a key role in T-cell and B-cell growth and development (Rastogi and Haldar, 2023); IL-10 is a multicellular and multifunctional cytokine, which can promote B-cell proliferation and antibody production (Asseman et al., 2003). TNF- α is an initiator of the inflammatory response that promotes the adsorption of leukocytes and vascular endothelial cells and enhances macrophage killing (Kisuya et al., 2019). In this study, we found that, with the extension of feeding time in a cold environment, the contents of IL-2 and IL-6 in the serum of sheep were significantly increased, while the contents of IL-10 were significantly decreased when CK aqueous extract was not added. This indicated that the immune performance of sheep was reduced and the immune-inflammatory response of sheep was activated under cold stimulation. Dietary additions of *Salvia sclarea* L. extract reduced the levels of the pro-inflammatory cytokines IL-1 β and TNF- α in lambs and increased the serum anti-inflammatory cytokine IL-10, thereby modulating the immune response (Ma et al., 2024). When *Astragalus membranaceus* root was added to the diet of Tibetan sheep, the concentration of serum IL (IL-2, IL-4, IL-10) increased with the increase in supplement concentration, enhancing the immune system (Wang et al., 2021). Studies have also shown that quercetin treatment in mice with septicemia (lipopolysaccharide-induced acute lung injury) can inhibit serum TNF- α , IL-1 β , IL-6, and NO secretion and increase IL-10 secretion (Gerin et al., 2016; Wang et al., 2014). In mice exposed to cold stress, treatment with kaempferol and cinnamon helped to inhibit the activated pro-inflammatory factors IL-9 and IL-13 and to increase the levels of the anti-inflammatory factors IL-2, IL-10, and IFN- γ (Jia et al., 2019; Yang et al., 2015). In this study, we found that the serum levels of IL-2, IL-6, and TNF- α were significantly lower and IL-10 was significantly higher in all groups of sheep fed diets supplemented with CK aqueous extract. This suggested that the addition of CK aqueous extract to the feed can result in a resistance to the development of immune-inflammatory responses caused by cold stimuli. The serum levels of IL-6 were the lowest and IL-10 levels were the highest when 8 $g\;{\mathrm{kg}}^{-1}$ CK aqueous extract was added, TNF- α levels were the lowest when 10 $g\;{\mathrm{kg}}^{-1}$ CK aqueous extract was added, and IL-2 levels were the lowest when 12 $g\;{\mathrm{kg}}^{-1}$ CK aqueous extract was added; these results indicate that there is a dose-dependent regulation of inflammatory factors in sheep organisms with the addition of CK aqueous extract.

In addition, serum immunoglobulins, as one of the indicators of humoral immune function, can bind to antigens and exert a variety of biological effects to stimulate antibody production (Yang et al., 2023b). IgG and IgM are the two major immunoglobulins in mammals (Schroeder and Cavacini, 2010). IgM plays a pioneering role against infection and is the main immunoglobulin in the early stages of anti-infection immunity in mammals; IgG is an important force in the fight against infection and is characterized by long maintenance, high levels, and wide distribution. In the present study, we found that the IgG and IgM levels in the serum of sheep in the group with no CK aqueous extract added were significantly reduced with the prolongation of rearing time in the cold environment, suggesting that the cold stimulation may inhibit the humoral immune function by affecting the synthesis of immunoglobulins. In contrast, the IgG and IgM contents in the serum of sheep in the group with the addition of CK aqueous extract were significantly higher, indicating that the addition of CK aqueous extract to the feed could result in a resistance to the suppression of humoral immune function caused by the cold stimulus. In particular, when 8 $g\;{\mathrm{kg}}^{-1}$ was added, the serum IgM and IgG contents were significantly increased, which further indicated that the addition of CK aqueous extract could better improve the immune performance of cold-stressed sheep.

Immune organs are places where immune cells such as lymphocytes, monocytes, macrophages, and granulocytes occur, differentiate, mature, settle, and proliferate, as well as carry out immune responses, and can be divided into central immune organs (e.g. the bone marrow, thymus, and avian bursa) and peripheral immune organs (e.g. the spleen, and lymphoid tissues). The thymus and spleen are the main reservoirs of T lymphocytes, which regulate the immune response and protect against pathogens and tissue damage (Khoso et al., 2016; Yang et al., 2016). Therefore, the present study investigated the effect of the addition of CK aqueous extract on the immune performance of the thymus and spleen of sheep. In the study, it was found that TNF- α , IL-2, and IL-6 contents in the spleen and thymus of sheep were increased, while IL-10, IgM, and IgG contents were decreased under cold-stimulation conditions without the addition of CK aqueous extract. In contrast, IL-2, TNF- α , and IL-6 contents in the spleen and thymus of sheep decreased, while IL-10, IgM, and IgG contents increased after the addition of CK aqueous extract; in particular, the effect was most obvious with the addition of 8–10 $g\;{\mathrm{kg}}^{-1}$ . These results suggest that cold stimulation may affect the normal functioning of immune organs and activate the immune-inflammatory response in the sheep organism by affecting cytokine levels and serum immunoglobulins. In contrast, the modulation of various immune factors in the body's immune organs by the addition of CK aqueous extracts had a dose-dependent effect, and the immunosuppressive ability of the spleen and thymus to resist the cold stimulus was most pronounced when the concentration of the added extracts was 8–10 $g\;{\mathrm{kg}}^{-1}$ .

In conclusion, adding a suitable amount of CK aqueous extract to the diet of sheep can improve their immune capacity under cold-stimulation conditions. When the additional amount is 8 $g\;{\mathrm{kg}}^{-1}$ , the effect is the best.

## 5 Conclusions

Cold stimulation will increase serum and liver lipid peroxides and reduce antioxidant capacity, resulting in oxidative stress in sheep. Adding an appropriate amount of CK aqueous extract into the feed can enhance the antioxidant capacity of sheep under cold conditions by improving the activity of antioxidant enzymes, increasing serum immunoglobulin, and regulating the secretion of various cytokines, thereby improving the immunity of sheep and then improving the growth performance of sheep. The recommended addition amount is 8 $g\;{\mathrm{kg}}^{-1}$ .

## Data Availability

Data will be made available upon reasonable request.
